# Density Functional Theory Insights into Polypyrrole-Based Functional Composites for Advanced Energy Storage, Sensing, and Environmental Applications

**DOI:** 10.3390/nano16050285

**Published:** 2026-02-24

**Authors:** Oluwaseye Samson Adedoja, Rendani Wilson Maladzhi, Oludolapo Akanni Olanrewaju, Samson Oluropo Adeosun, Oluwatoyin Joseph Gbadeyan

**Affiliations:** 1 Department of Mechanical Engineering, Durban University of Technology, Durban 4000, South Africa; rendanim1@dut.ac.za; 2 Institute of System Science, Durban University of Technology, Durban 4000, South Africa; oludolapoo@dut.ac.za (O.A.O.); toyin2good@gmail.com (O.J.G.); 3 Department of Metallurgical and Materials Engineering, University of Lagos, Akoka 101017, Lagos, Nigeria; sadeosun@unilag.edu.ng; 4 Department of Industrial Engineering, Durban University of Technology, Durban 4000, South Africa; 5 Engineering Technology, Collin College, Plano, TX 75069, USA

**Keywords:** DFT, computational materials design, energy storage, dopant–polymer interactions, polypyrrole composites, functional composites, environmental applications, charge transfer behavior

## Abstract

Polypyrrole-based functional composites are increasingly explored and extensively adopted for energy storage, sensing, and environmental applications due to their tunable electronic properties, chemical versatility, and mechanical stability. However, rational optimization of these composites requires a unified understanding of electronic, mechanical, thermal, and chemical behavior at the atomic scale, which underlies their multifunctional behavior, and remains fragmented. Notably, Density Functional Theory (DFT) provides indispensable atomistic insight into the electronic, mechanical, thermal, and chemical interactions that govern the performance of multifunctional materials. To bridge these gaps, this review presents a comprehensive assessment of recent DFT and time-dependent DFT (TD-DFT) studies that elucidate the electronic, mechanical, thermal, and chemical characteristics of polypyrrole and its hybrid composites. Key theoretical descriptors, including electronic structure modulation, charge transfer behavior, adsorption energetics, interfacial binding energies, hydrogen bond formation, and charge redistribution, are critically assessed to establish structure–property relationships across diverse functional systems. Considerable attention is given to interfacial interactions, doping strategies, and composite architectures that govern durability, conductivity, and chemical stability. By consolidating current atomistic insights and identifying existing limitations, this review provides a coherent framework for rational material design. Notably, it presents the first systematic quantification of dopant steric effects in PPy multifunctional composites, linking atomistic-scale modifications to the optimization of functional properties in next-generation applications.

## 1. Introduction

Conducting organic polymers (COPs), characterized by their π-conjugated backbones, represent a class of technologically significant materials due to their tunable electronic properties, low cost, and facile synthesis [1,2,3,4,5,6,7,8,9,10,11]. Among these, polypyrrole (PPy), a heteroatomic polymer containing nitrogen within its five-member ring, has garnered considerable attention due to its high electrical conductivity, environmental stability, and superior redox characteristics [4,12,13,14,15,16,17]. These features make PPy a promising candidate for a wide range of applications, including electronic devices, solar cells, supercapacitors, and chemical sensors (see Table 1) [1,18,19,20,21,22,23].

In its natural state, PPy typically behaves as an insulator, exhibiting a relatively wide experimental band gap (Eg) of approximately 2.85–3.20 eV [20,24]. However, its intrinsic conductivity can be reversibly modulated through oxidation and reduction processes, commonly referred to as doping and dedoping [2,18]. During oxidation (p-doping), positive charge carriers such as polarons (radical cations) and bipolarons (dications) are generated along the polymer backbone, introducing new electronic states within the band gap. This leads to an insulator-to-metal transition essential for high conductivity [18,25,26]. The practical implementation of PPy in advanced functional materials relies critically on the precise control of its structural and electronic interactions, especially when forming nanocomposites with other active phases [27,28].

These interfacial processes and charge carrier dynamics require computational tools capable of resolving electronic structure at molecular resolution. These computational tools, ranging from wavefunction-based quantum chemistry methods to density functional and many-body approaches, enable molecular-level resolution of electronic structure, providing a unified framework for predicting structure–property relationships across molecules, interfaces, and extended materials. At the core of modem electronic structure modeling, DFT has become central to this task, providing access to optimized geometries [1,2,29,30,31,32,33,34,35,36], frontier molecular orbital energies [1], band gaps [37,38], ionization potentials, and electron affinities [1,2,25]. Time-dependent DFT (TD-DFT) extends these insights to optical activity [1,18]. Additional analyses such as quantum theory of atoms in molecules [39] and reduced density gradient methods [20,40] reveal non-covalent interactions, hydrogen bonding, and π-stacking effects that govern composite stability [20,41].

**Figure 1:**
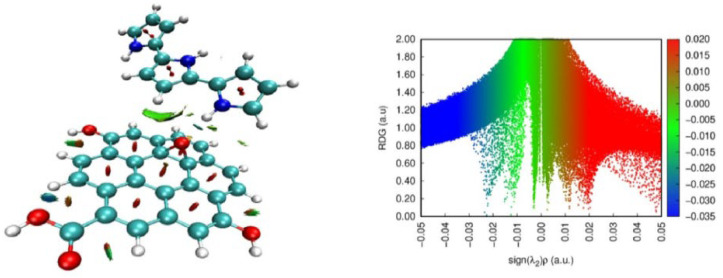
Optimized geometries and interfacial interaction analysis of PPy nanocomposites. Reduced density gradient (RDG) isosurfaces for a PPy–graphene complex, color-coded (blue = attractive, green = vdW, red = repulsive) to confirm π–π and lone-pair–π interactions at the interface [39].

Interfacial mechanisms represent a fundamental part of PPy composite behavior. For PPy adsorbed on the Fe_2_O_3_(104) surface, a stabilizing hydrogen bond of 1.57 Å accompanies an adsorption energy of −2.10 eV, indicating strong interface formation, as shown in Figure 4e of Ref. [42]. When PPy interacts with carbonaceous substrates, van der Waals π–π stacking dominates on pristine graphene, yet the interaction becomes stronger in the presence of structural defects or oxygenated groups. Epoxy-functionalized graphene oxide (GO_3_) enhances the adsorption energy to −28.79 kcal mol^−1^. Charge density difference maps clarify the redistribution of electronic charge in PPy/oxide and PPy/carbon composites (see Figure 1b of Ref. [32]), while reduced density gradient isosurfaces distinguish attractive, dispersive, and repulsive interaction regions and highlight the coexistence of π–π and lone-pair interactions at PPy interfaces (Figure 1) [39,40].

**Figure 2:**
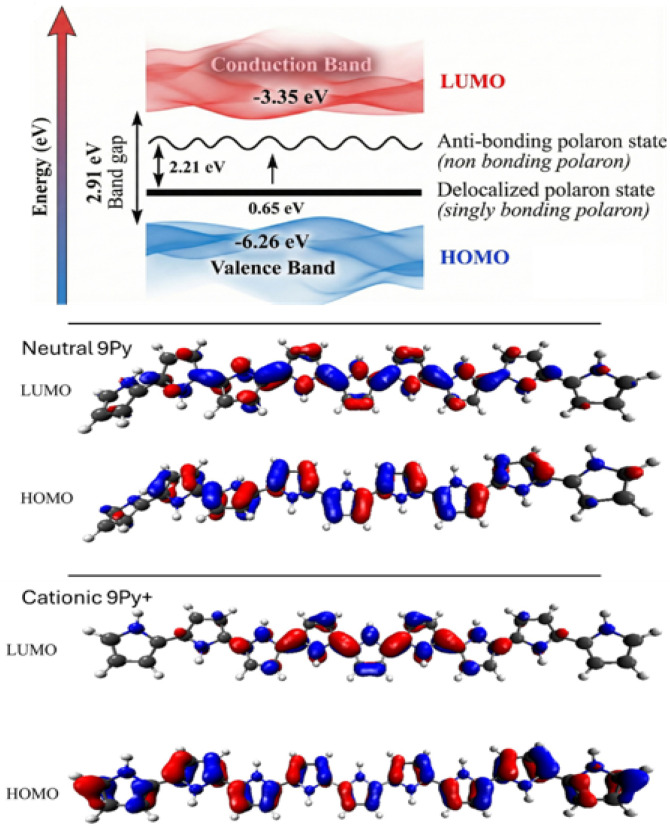
DFT analysis of electronic structure tuning via doping and functionalization. Energy-level diagram illustrating HOMO–LUMO gap (Eg) reduction upon p-doping: neutral 9Py (Eg = 3.41 eV) vs. cationic 9Py^+^ (Eg = 2.91 eV) [2].

**Figure 3:**
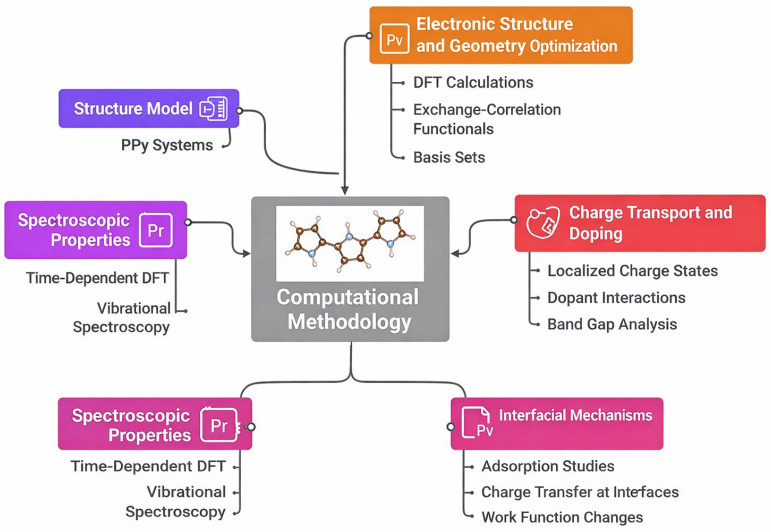
Illustration of computational methodology for PPy systems. Optimized geometry workflow: representation of elongation and geometric optimization process applied to PPy oligomers [55].

Beyond these interfacial considerations, electronic structure modulation within doped or hybrid PPy systems is equally important. Oxidation of a nine-unit PPy chain (9Py → 9Py^+^) reduces its band gap from 3.41 eV to 2.91 eV (Figure 2). Frontier Molecular Orbitals (FMOs) of PPy oligomers interacting with formaldehyde (CH_2_O), showing HOMO/LUMO localization changes upon adsorption (see Figure 3a–f of Ref. [43]). These electronic modulations also govern sensing behavior. The integration of formaldehyde during or after polypyrrole synthesis aids chemical cross-linking via methylene (–CH_2_–) bridges between pyrrole units, resulting in a cross-linked conductive polymer network. This structural modification boosts mechanical stability, influences charge transport behavior, and regulates morphological features, including porosity and chain packing. Thus, formaldehyde-modified polypyrrole exhibits improved performance in various functional applications, including sensors, protective coatings, and energy storage devices. Formaldehyde produces a strong perturbation on an anionic PPy chain, with an interaction energy of −31.37 kcal mol^−1^ and an increase in the band gap from 0.40 eV to 1.85 eV, corresponding to a sensitivity of 362.5 percent (see Figures 3a and 4b,d of Ref. [43]).

Computational methods have also clarified solvent effects and co-doping strategies in sensing applications. Nitrate interactions shift from −20.2 kcal mol^−1^ in the gas phase to −7.35 kcal mol^−1^ in aqueous media [3]. PPy doped with dodecyl benzene sulfonate improves NO_2_ adsorption from −0.511 eV to −0.676 eV and increases charge transfer from 0.411 |e| to 0.521 |e| [44]. In electronic and optoelectronic contexts, heteroatom substitution offers further tunability. Si-doped PPy provides effective band gap reduction, [45] and electron-withdrawing substituents such as NO_2_ enhance stability [20]. Polaronic PPy structures support strong nonlinear optical behavior, with the first hyperpolarizability rising from 1.3 × 10^2^ au in neutral 9Py to 3.2 × 10^4^ au in 9Py^+^ [25].

This review consolidates recent DFT and TD-DFT investigations, highlighting the fundamental mechanisms governing charge transport, interfacial stability, and property modulation in PPy-based composites for energy storage, sensing, and environmental applications. To ensure a comprehensive review analysis, this work adopts a systematic approach and conducts searches across prominent journal databases, including Web of Science, Scopus, IEEE Explore, ScienceDirect, and Google Scholar. Keywords include combinations of “polyprrole”, “conducting polymer”, “density functional theory”, “ DFT”, “composite”, “energy storage”, “sensing”, and “ environmental applications” using Boolean operators to refine results. The search focuses on peer-reviewed original research and high-quality computational studies with clearly defined methodologies, published between 2010 and 2025, to capture recent advances without compromising relevance. The search excludes conference abstracts and reports that lack appropriate computational and experimental details. This systematic approach is adopted in this study to ensure robust coverage of the review processes.

## 2. Computational Methodologies and Mechanistic Interpretation

The successful deployment of polypyrrole (PPy) in functional composites requires a deep, atomic-level understanding of its electronic structure, charge transport mechanisms, and complex interfacial interactions (Figure 3). Density Functional Theory (DFT) has emerged as the principal computational method utilized for optimizing and predicting these properties, balancing accuracy with feasibility, especially for large polymeric and composite systems [33,35,37,46,47]. Methodological choices regarding the exchange–correlation (XC) functional and basis set are critical for accurately capturing PPy’s distinctive chemical physics (see Table 2) [3,43]. Hybrid functionals, particularly the Becke three-parameter Lee–Yang–Parr (B3LYP) functional, are widely favored for investigating the geometric, spectroscopic, and electronic characteristics of PPy oligomers [1,35,48,49,50]. When modeling charged species or open-shell systems like polarons, the Unrestricted B3LYP (UB3LYP) formalism is frequently employed [2,50]. For solid-state or periodic systems, the Generalized Gradient Approximation (GGA), typically using the Perdew–Burke–Ernzerhof (PBE) functional, is standard within packages like VASP and CASTEP [24,33,51,52,53,54]. Basis sets generally include the Pople-style sets such as 6-31G(d), incorporating polarization functions on non-hydrogen atoms for improved accuracy in geometry and electronic structure calculations [40,43,49,50,55].

Given that PPy composites heavily rely on non-covalent forces such as π–π stacking and van der Waals (vdW) interactions, the incorporation of dispersion corrections is essential to avoid underestimation of binding energies [3,43]. Grimme’s DFT-D3 correction is widely adopted in both molecular (Gaussian) and periodic (VASP) calculations [40,52,56,57]. Alternatively, long-range corrected functionals, such as the M06-2X meta-hybrid functional [40] and ωB97X-D [18], are used, often demonstrating improved performance in quantifying binding affinity [18]. Samanta and Das [40] reported that utilizing the M06-2X-D3 method resulted in a relative root-mean-square deviation of 2.2 kcal mol^−1^ in total binding energies for PPy/graphene complexes, significantly better than the 5.3 kcal mol^−1^ deviation found using M06-2X without D3 correction. Various Software packages frequently utilized in these studies include Gaussian 09/16 for molecular cluster calculations, [2,25,37,52,57,58] VASP (Vienna Ab initio Simulation Package) [52,53,54,59,60] and CASTEP [21,51,61] for periodic simulations, and DMol^3^ for calculations involving numerical basis sets [21,62,63].

Geometry optimization is the foundational step, verifying true energy minima (absence of imaginary frequencies) and revealing the relationship between chemical environment and molecular conformation [2,35,64]. Neutral pyrrole oligomers (nPy) typically exhibit a non-planar conformation characterized by anti-gauche torsion angles between adjacent rings, often reported in the range of 151–160° [40,43]. Doping, such as p-doping (oxidation), dramatically alters the molecular structure; Ullah et al. found that the polaron state 9Py^+^ is planar [2]. This planarization enhances the superposition of π orbitals, thereby increasing polymer conductivity [64]. In a PPy composite structure, the mean dihedral angle decreased sharply from 64.0° (free PPy) to 6.3°, demonstrating increased π-orbital overlap [64]. DFT systematically models the evolution of the Frontier Molecular Orbitals (FMOs), HOMO and LUMO, and the resultant band gap (E_9_) with increasing chain length (n) [1,18,58]. The electronic properties derived from oligomers (up to n = 9) are frequently extrapolated to the infinite polymer (∞Py) using a second-order polynomial fit, validating the use of finite models [2,35,43]. The extrapolated E_9_ value for ∞Py calculated using B3LYP/6-31G(d) is 2.88 eV [43], closely aligning with experimental values. Computational modulation via substituents, such as the push–pull set OCH_3_ and NO_2_ in [OCH_3_-(PPy)_4_-NO_2_], successfully reduced the band gap to 2.8 eV [20,35].

DFT is crucial for quantifying adsorption and interfacial interactions (E_a_d_s_ or E_int_), which govern the functionality of PPy nanocomposites. The interaction energy is commonly calculated using the formula described in Equation (1) [40,42,57]:

E_ads_ = E_complex_ − (E_substrate_ + E_PPy_) (1)

For PPy physisorbed on pristine graphene, the M06-2X-D3 adsorption energy was determined to be approximately −25 kcal mol^−1^ [40]. The presence of defects or functional groups significantly amplifies this non-covalent binding strength; the introduction of epoxy groups on graphene oxide enhanced the binding energy to −34 kcal mol^−1^ [40]. In sensing studies, the counterpoise (CP) correction is often applied to the interaction energy (E_int_) calculation to mitigate the basis set superposition error (BSSE), providing ΔE_int_ CP [43,49,57]. For the interaction of pyrrole oligomers with small analytes, the B3LYP-D3(BJ)-CP/6-31+G(d,p) method yielded an E_int_ = −5.74 kcal mol^−1^ for the 1Py–CH_2_O complex [57]. The calculated interaction energies for systems like 3PPy–CH_4_ show the necessity of BSSE correction, resulting in a minimal binding energy of only 0.11 kJ mol^−1^ [35,49].

Charge transfer analysis elucidates the redox chemistry and doping behavior essential for PPy’s electronic applications. Mulliken [1,2,50] and Natural Bond Orbital (NBO) [1,2,50] methods are routinely used to quantify charge distribution across the polymer and the composite component [50]. Additionally, Bader’s Quantum Theory of Atoms in Molecules (QTAIM) is employed to analyze electron density topology, precisely defining atomic interactions and charge transfer [18,47,65]. In PPy/SDBS-PF adsorption models, Bader’s theory quantified the charge gained by the PF molecule to be between 0.40–0.56 |e| [66]. DFT studies confirm that PPy generally behaves as a p-type material, donating electron cloud density to inorganic constituents like TiO_2_ in nPy–TiO_2_ composites [67].

Advanced analytical tools further interpret mechanistic subtleties. Total and projected density of states (TDOS/PDOS) plots map the available electronic states and quantify orbital hybridization, linking electronic structure directly to electrochemical activity [20,51,52,67,68]. The presence of significant orbital overlap near the Fermi level in MMT/PPy composites suggests facilitated electron transfer, promoting a transition toward a metallic state and enhancing electroactivity [51]. Molecular Electrostatic Potential (MESP/ESP) mapping graphically identifies regions susceptible to electrophilic or nucleophilic attack, guiding the prediction of active sites for gas sensing [21,69,70]. Furthermore, advanced non-covalent interaction tools, such as reduced density gradient (RDG) analysis, provide spatial visualization of weak forces (e.g., π–π stacking, hydrogen bonds, and lone-pair–π interactions) governing the integrity of PPy nanocomposites [40]. Finally, condensed-to-atoms Fukui indices are calculated to systematically predict the local reactivity and optimal adsorption centers of PPy derivatives toward toxic gases like Cl_2_ and SO_2_ [70].

## 3. Structure–Property Relationships

The intrinsic functionality and high tunability of polypyrrole (PPy) stem directly from its unique chemical structure, which allows for dynamic structural and electronic transitions in response to external stimuli or incorporation into composites. PPy belongs to the family of conjugated organic polymers (COPs), characterized by alternating single and double bonds along the polymer backbone, which enables a system of delocalized π-electrons beneficial for electrical and optical transport [1,50,71]. In its neutral state, PPy typically exhibits a non-planar conformation [2,43], rendering it an insulator with a wide electronic band gap (Eg) ranging from approximately 2.85 eV to 3.20 eV [20]. Density Functional Theory (DFT) calculations, often relying on extrapolation from oligomer studies (up to n = 20) to the infinite chain, support this, yielding estimated Eg values of 2.88 eV for native PPy [35,37].

### 3.1. Doping, Conformation, and Charge Carrier Dynamics

**Figure 4:**
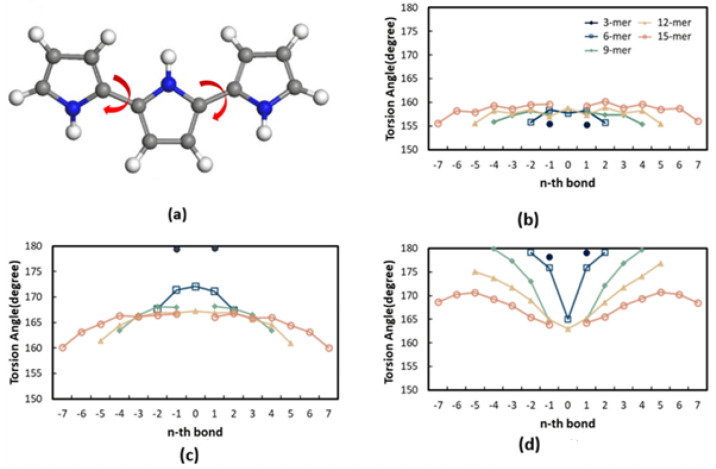
Molecular structure and conformational changes of polypyrrole (PPy). Representation of structural transitions reflecting changes in intermonomer torsion angles as (**a**) PPy evolves from (**b**) neutral state to (**c**) polaronic (radical cation, S = 1/2) or (**d**) bipolaronic (dication, S = 0) state [41].

The electrical conductivity (σ) of PPy is highly correlated with its oxidation state and resulting geometry, defining the core structure–property paradigm. The insertion of positive charges (p-doping) transforms the material from an insulator (benzenoid structure) into a conductor (quinoid structure) [18,26,55]. This oxidation process generates polarons (radical cations, S = 1/2) and bipolarons (dications, S = 0) [18,26,72]. DFT modeling reveals that the polaron state (9Py^+^) in Figure 4 adopts a planar geometry, increasing π-orbital superposition, which intrinsically enhances polymer conductivity [2,25,43]. This planarization effect is quantitatively confirmed in composites, where the mean dihedral angle of PPy decreases sharply from 64.0° (free PPy) to 6.3° upon incorporation into a PPy/C_3_N_4_ composite [64]. This geometric change minimizes energy loss during ion intercalation, a key charge storage mechanism for PPy [64]. Concomitantly, the intermonomer single-bond lengths decrease, and the intramonomer double-bond lengths increase as the chain advances from the neutral to the polaronic and further to the bipolaronic state (Figure 4a–d) [41].

The HOMO–LUMO gap narrows under p-doping conditions (e.g., from 3.41 eV for neutral 9Py to 2.91 eV for 9Py^+^), which facilitates charge transport. The introduction of mid-gap electronic states by polarons and bipolarons is central to the conductivity transition. The lowest theoretical band gap reported for PPy corresponds to the n-type-doped infinite chain (∞Py^−^), at approximately 0.40 eV, which is far smaller than those observed under p-type doping.

DFT calculations provide atomic-level correlation between structure and electronic behavior. Upon oxidation of 9Py to 9Py^+^ (polaron formation), the band gap is significantly reduced by 0.51 eV, from 3.41 eV to 2.91 eV [2]. The presence of polaron states introduces new energy levels within the band gap, facilitating electronic transitions [2]. For the highly conductive 9Py^+^ species, a prominent band corresponding to a delocalized polaron is identified at 0.65 eV. This delocalized polaron is responsible for both lower resistance and higher conductivity.

The incorporation of functional groups or atomic dopants modulates these electronic properties. For instance, substituent effects show that attaching electron-donating groups such as –OCH_3_ or –OH to PPy leads to improved optical properties, producing a redshift greater than 20 nm relative to unmodified PPy [20]. Doping effects, as studied by Mariyappan and Gnanasekaran, demonstrate that heteroatom substitution at the nitrogen sites significantly alters the electronic landscape, with silicon-doped PPy exhibiting the most pronounced band gap narrowing, indicating enhanced intrinsic conductivity suitable for sensing and electronic applications [45]. Natural bond orbital (NBO) and Mulliken population analyses confirm that p-type dopants facilitate charge transfer from the polymer, while n-type dopants induce charge transfer to the polymer [2]. The absolute values of the calculated electronic descriptors, particularly for charged and radical cationic PPy species, are dependent on the choice of exchange correlation functional. On the other hand, the Generalized-Gradient Approximation-based approaches tend to underestimate ionization potentials, band gaps, and charge localization, whereas hybrid functionals typically produce more reliable quantitative estimates, while preserving the same qualitative trends in the reactivity and interfacial charge transfer behavior. For example, the 9Py^+^ polaronic state exhibits a reduced ionization potential (IP) compared to neutral PPy, with IP values decreasing monotonically with increasing chain length (conjugation) [25]. This reduction in IP and the smaller HOMO–LUMO gap are correlated with a drastic enhancement in static hyperpolarizability (β_0_): the 9Py^+^ polaron state exhibits a β_0_ value 246 times higher (3.2 × 10^4^ atomic units) than its neutral analogue (1.3 × 10^2^ atomic units), positioning PPy polarons as promising nonlinear optical (NLO) materials [25].

### 3.2. Interfacial Interactions in PPy Composites

The performance enhancement of functional PPy composites relies critically on optimizing interfacial electronic coupling and charge transfer pathways between the polymer and inorganic fillers (Table 3). DFT calculations incorporating dispersion corrections (for example, the M06-2X-D3 functional) are essential for characterizing these interactions, which are typically governed by weak non-covalent forces [40].

#### 3.2.1. Carbon Nanomaterials (Graphene, CNTs, and Carbon Fibers)

The binding of PPy onto pristine graphene (G) is primarily a physisorption process driven by π–π stacking and van der Waals (vdW) interactions, with a typical adsorption energy of approximately −25 kcal mol^−1^ [40]. Functionalization dramatically enhances this interaction; for example, the introduction of epoxy groups on graphene oxide (GO_3_) increases the binding affinity to –34 kcal mol^−1^ [40]. The hybridization of PPy with fillers such as multi-walled carbon nanotubes (MWCNTs) improves electronic transport, as confirmed by a significant reduction in the composite’s optical band gap from 2.89 eV (PPy) to 1.58 eV (PPy/MWCNTs composite) [21]. This band gap narrowing results in increased direct-current electrical conductivity (σdc), reaching values up to 56.08 × 10^−5^ S m^−1^ [21]. Strong interactions between the highly delocalized π-electrons of the CNTs and those of the PPy skeleton favor electron and hole transfer, resulting in faster electrode kinetics [19].

#### 3.2.2. Metal Oxides, Nanodiamonds, and MXenes

In hybrid systems with inorganic substrates, DFT analyses often highlight favorable heterojunction formation and efficient charge separation. For instance, in PPy/nanodiamond (ND) composites, the highest occupied molecular orbital (HOMO) is localized on PPy while the lowest unoccupied molecular orbital (LUMO) is localized on ND [64,73,74], leading to spatial separation that is energetically favorable for exciton dissociation in photovoltaic applications [64]. In montmorillonite (MMT)/PPy composites, strong interfacial coupling (adsorption energy of −2.56 eV) is stabilized by hydrogen bonding between PPy N–H groups and oxygen atoms on the MMT surface. DFT confirms that PPy acts as an electron donor, transferring approximately 0.41 electrons to the MMT layer, establishing a built-in electric field that enhances cation capture, such as Pb^2+^ adsorption [51]. For PPy/SnS thermoelectric composites, the incorporation of SnS nanoparticles into the PPy matrix increases the total density of states near the Fermi level (EF) due to C 2p and S 3p orbital overlap, reducing the band gap from 2.25 eV to 1.03 eV and improving the thermoelectric power factor [52]. Similarly, in PPy/MXene systems, DFT models show clear electron transfer from MXene to PPy, resulting in a higher density of states near EF and thus improved electronic conductivity [75].

### 3.3. Morphology-Dependent Functionality and Performance

The deliberate design of PPy morphology, from continuous films to porous nanostructures, is a critical strategy for enhancing its performance across energy storage, sensing, and environmental remediation applications [48,76].

For electrochemical applications, nanostructured PPy such as nanotubes or thin films is preferred for pseudocapacitors because the effective ion diffusion length (approximately 40 nm) is well accommodated by nanostructured architectures, allowing for full utilization of the active material during charge–discharge cycles [77]. The morphology also affects specific surface area: for instance, sodium dodecylbenzene sulfonate (SDBS) functionalization significantly reduces the PPy nanosheet diameter from 365 nm (pure PPy) to 148 nm, resulting in a larger specific surface area and improved electrochemical activity [67].

Cycling stability, a common limitation in PPy due to swelling and shrinkage during redox cycling, is substantially improved through composite formation [42,77]. Strong adsorption between PPy and substrates such as carbon fiber (CF) or MMT (with adsorption energies up to 45.53 kJ mol^−1^) helps restrict volume expansion and thus enhances long-term stability [51,78]. Similarly, PPy coatings on zinc nanowire arrays improve stress distribution and structural integrity during operation [42].

Morphology also affects adsorption and sensing behavior. Porous or nodular PPy structures increase the electroactive area and accessibility of active sites for analyte binding. In MMT/PPy composites, the incorporation of PPy increases interlayer spacing within MMT and expands the interfacial region, thereby enhancing Pb^2+^ adsorption energy to 4.81 eV. DFT calculations indicate that this structure provides quasi-one-dimensional diffusion pathways, significantly improving ion migration kinetics.

## 4. PPy in Energy Storage Systems

Polypyrrole (PPy) possesses intrinsic electronic conductivity and tunable electrochemical activity, making it a key material for high-performance electrodes in modern energy storage systems, particularly rechargeable metal-ion batteries (MIBs) and supercapacitors (SCs) [79]. Pristine PPy exhibits a reversible lithium storage capacity of 72 mAh g^−1^ above 2.0 V versus Li^+^/Li. However, PPy is most commonly employed as a conductive matrix, surface coating, or host component in nanocomposites designed to mitigate intrinsic limitations of active materials, including volume expansion, dissolution, and sluggish ion transport kinetics [. For example, PPy coatings applied to materials such as LiFePO_4_, V_2_O_5_, and Sn significantly enhance rate capability and cycling stability [79]. Wu and co-workers further demonstrated that PPy effectively suppresses the dissolution of electrode materials such as V_2_O_5_ and LiV_3_O_8_ in aqueous rechargeable lithium batteries, leading to improved long-term cycling behavior [79]. Density Functional Theory (DFT) provides crucial insights into the interfacial mechanisms responsible for these improvements, particularly regarding ion adsorption, charge redistribution, and diffusion behavior [79].

In the context of next-generation MIBs, including sodium-ion batteries (SIBs) and potassium-ion batteries (PIBs), PPy-based composites are essential for enabling high specific capacities and long-term cycling stability [79]. For SIB anodes, DFT calculations by Ezika et al. on Ti_2_CO_2_^−^ MXene/PPy nanocomposites predicted a narrow electronic band gap of 0.02 eV and a favorable sodium-ion adsorption energy of −0.44 eV [4]. Charge transfer analysis indicated that physisorption dominates Na-ion binding, with sodium atoms adsorbing at distances of 2.95 Å from the MXene surface and 2.60 Å from the PPy layer. Zeng et al. further demonstrated that porous PPy/carbon anodes deliver an ultrahigh capacity of 552 mAh g^−1^ at 0.1 A g^−1^, maintaining 86.5% capacity after 200 cycles [80]. Complementary DFT results indicated that the synergistic electronic interactions between porous carbon and PPy enhance Na anchoring by optimizing charge transfer pathways [80]. For SIBs and PIBs, Yi et al. reported that VSe_2_@PPy nanoplates retain 324.6 mAh g^−1^ after 2800 cycles at 4 A g^−1^, with DFT confirming that strong heterointerfacial coupling produces a built-in electric field, increases the total density of states (DOS), reduces the ion diffusion barrier, and improves electronic conductivity [81]. In the lithium–sulfur system, work by Fu and Manthiram on S–PPy core–shell cathodes revealed that the α-ZrP/PPy heterostructure exhibits a low Li_2_S decomposition barrier of 0.54 eV, demonstrating enhanced redox kinetics facilitated by electronic-channeling effects within the PPy matrix [82,83].

PPy composites have also shown exceptional performance in stabilizing zinc metal anodes in aqueous zinc-ion batteries (ZIBs), particularly by suppressing dendrite formation. DFT simulations by Adedoja et al. demonstrated that the adsorption of PPy onto graphene is strongly exothermic, with an adsorption energy of −1.68 eV, yielding a structurally stable G/PPy interface [46]. The maximum zinc intercalation capacity predicted for the composite was 510.12 mAh g^−1^, while the Zn adatom diffusion barrier was extremely low at 12 meV, indicating excellent ion transport kinetics. Experimentally, PPy nanorod-coated zinc anodes have shown stable cycling over more than 540 cycles, while PPy/reduced-graphene-oxide (rGO) coatings similarly suppress dendrite formation. Li and co-workers further demonstrated that PPy/MnO_2_ layers enhance Zn^2+^ diffusion through a combination of spatial confinement and favorable electronic interactions. Baek et al. reported that metal-ion doping significantly improves the conductivity of PPy, with Zn-doped PPy exhibiting a reduced band gap of 0.35 eV, relative to 1.77 eV for undoped PPy [46].

In supercapacitor applications, PPy functions as a pseudocapacitive material in which charge is stored through rapid, reversible ion doping and dedoping processes [81]. A representative case is the C_3_N_4_/PPy composite supercapacitor, which exhibits markedly enhanced electrochemical performance [64]. The GCD curves in Figure 4a,b in Ref. [64] show that the PPy+C_3_N_4_ composite attains a mean specific capacitance of 810 F g^−1^ at a current density of 0.20 A g^−1^, a substantial improvement over the 204 F g^−1^ recorded for pristine PPy. The composite also preserves its performance under high-current conditions, retaining 610 F g^−1^ at 9.0 A g^−1^, which highlights its operational stability. This elevated capacitance is partly linked to the enlargement of the electro-active surface area that arises from the development of a more porous morphology. Electrochemical Impedance Spectroscopy, typically displayed as Nyquist plots (as indicated in Figure 3e of Ref. [84]), further demonstrates that the composite possesses a significantly reduced charge transfer resistance (Rct), reflecting strengthened structural integrity and improved ion diffusion. Supporting DFT calculations indicate that hydrogen bonding plays a critical stabilizing role, lowering the H-bond energy by 46.56 kJ mol^−1^ and promoting the robustness of the composite network [65,84].

DFT and time-dependent DFT (TD-DFT) analyses clarify how these redox processes influence electronic structure. Ullah et al. showed that p-doped PPy, modeled at the UB3LYP/6-31G(d) level, exhibits a reduced band gap, enhanced conductivity, and stronger π–electron conjugation [2]. Additional DFT studies have shown that interactions between PPy and carbon nitride reduce energy losses during ion intercalation [64]. PPy-based hybrids often employ carbonaceous scaffolds to enhance structural stability and electron transport. For example, PPy deposited on carbon foam produces a significant capacitance increase from 37.3 mF cm^−2^ to 364.0 mF cm^−2^, while retaining 99.6% of its capacity after 10,000 cycles [82]. Arias-Pinedo et al. demonstrated that CF–rGO–PPy electrodes exhibit specific capacitances of 742.5 F g^−1^ (CV) and 905 F g^−1^ (GCD), with approximately 96% retention after 10,000 cycles [72]. TD-DFT analysis of this system revealed distinct polaronic transitions at 550.31 nm (T_1_) and 1041.09 nm (T_2_), corresponding to a net +1 charge state [73] The interface stability of PPy composites is further supported by DFT studies from Samanta and Das, who reported strong PPy adsorption on graphene oxide surfaces, with adsorption energies of −28 kcal mol^−1^ at Stone–Wales defects and −34 kcal mol^−1^ at epoxy functional groups, compared to −25 kcal mol^−1^ on pristine graphene [84]. DFT simulations of PPy/multi-walled carbon nanotube (MWCNT) composites additionally predict an optical band gap of 1.58 eV, significantly lower than the 2.89 eV characteristic of pure PPy [85].

Hybrid supercapacitor electrodes combining PPy with transition metal compounds or MXene materials further demonstrate enhanced electrochemical stability. Du et al. fabricated multilayer MXene-stabilized PPy composites (MXene@PPy) that deliver a specific capacity of 124.9 mAh g^−1^ at 1.0 mA cm^−2^, retaining approximately 80.3% of capacity over 2500 cycles [75]. DFT calculations confirmed significant electron transfer from MXene to PPy, generating electron-rich regions and increased DOS that enhance redox activity [75]. Vigneshwaran et al. developed a V_2_C–PPy–PdO ternary composite exhibiting a specific capacitance of 487 F g^−1^ at 1 A g^−1^, with 92% retention after 10,000 cycles; DFT analysis attributed this performance to PdO-induced increases in electronic states near the Fermi level, improving quantum capacitance [85]. In capacitive deionization (CDI), PPy also enhances deionization capacity, with PPy/graphene-oxide composites showing particularly strong ion removal efficiency.

### Treatment of Solvation Effects in DFT Studies of Energy Storage Systems

Electrolyte solvation plays a crucial role in governing interfacial stability, ion transport, and charge transfer kinetics in electrochemical energy storage systems. Still, a good number of DFT works on electrode materials and redox-active players are influenced by either gas-phase or vacuum-based approximations, which exclude solvent screening, electrolyte polarization, and ion–solvent coordination effects [86]. Although these approaches are important for establishing fundamental electronic structure and qualitative adsorption tendencies, their predictive relevance under battery conditions is limited due to their frequent overestimation of binding energies, misplacement of reaction barriers, and failure to adequately account for electrode–electrolyte reactions [87]. To overcome these challenges, researchers have extensively embraced implicit solvation models such as the Polarizable Continuum Model (PCM), Conductor-like Screening Model (COSMO), and Vienna Ab initio Simulation Package implicit SOLvation model (VASPsol) in battery-related DFT studies [88,89]. These approaches offer a better estimate of electrochemical properties at a moderately low computational cost by considering the electrolyte as a homogenous dielectric medium [90]. However, certain solvent–solute reactions, hydrogen binding, ion pairing, and local structural heterogeneity—all of which are crucial at electrified interfaces and during the manufacturing of solid–electrolyte interphase (SEI)—are intrinsically excluded by implicit approaches [91,92].

On the other hand, explicit solvation approaches incorporate solvent molecules and mobile ions directly into the simulation cell, giving a substantially more accurate characterization. In addition, it facilitates the dynamic sampling of solvation shells, the electric double-layer structure, and early-stage electrolyte decomposition pathways when combined with ab initio molecular dynamics (AIMD) [93]. Although these approaches have provided significant insights into SEI chemistry and interfacial charge transfer, their applicability for large-scale material screening is constrained by their computational cost, limited accessibility, and sensitivity to both system size and sampling statistics. To strike a balance between computing efficiency and physical plausibility, hybridization of explicit–implicit solvation approaches has emerged. These techniques are becoming increasingly attractive for DFT studies of batteries and polymer-based electrode composites because they have demonstrated enhanced accuracy in modeling electrochemical energetics and interfacial stability while preserving adaptable computational costs [94]. Even with these technological developments, it remains challenging to accurately describe electrochemical surfaces quantitatively within classical DFT models, especially when operating under dynamic conditions. Thus, new approaches such as grand-canonical formulations, constant-potential DFT, and machine learning-assisted solvation sampling are drawing interest from researchers as viable paths towards more accurate, experimentally applicable simulations of electrolyte–electrode interfaces in next-generation energy storage systems [95].

## 5. Sensing and Detection

Polypyrrole (PPy) and its composites have emerged as highly effective materials for chemical, electrochemical, and biological sensing owing to PPy’s tunable conductivity, large specific surface area, environmental stability, and redox-active nature in both its neutral and doped states [3,18,50,96]. DFT calculations play a central role in elucidating the molecular origins of sensing performance by characterizing adsorption geometries, charge transfer (CT) processes, and electronic structure modulation. These computational insights directly inform experimental strategies to improve selectivity and sensitivity across gas-phase, electrochemical, and biosensing applications [57,96].

### 5.1. Gas Sensing Mechanisms and Selectivity

PPy-based materials are widely used as chemiresistive gas sensors, where analyte adsorption induces measurable changes in conductivity and band structure [50,57]. DFT studies consistently show strong interactions between PPy and polar or oxidizing gases. For example, in $NH_{3}\;$detection, both neutral PPy (nPy) and doped PPy ($PPy^{+}$) exhibit significantly stronger binding than for $CO_{2}$ or CO detection [50]. Ullah et al. reported BSSE-corrected $NH_{3}$ adsorption energies on neutral PPy oligomers ranging from −9.98 to −10.53 kcal ${mol}^{-1}$, attributed to strong hydrogen bonding [29,50]. These interactions reduce the dihedral angle ∠C1C2N3C5, lowering the resistance to electron movement along the backbone and increasing conductivity [29].

**Figure 5:**
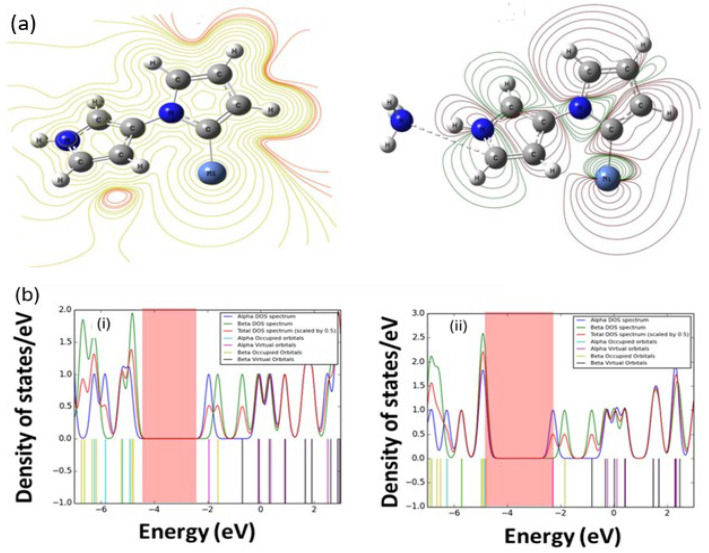
DFT analysis of gas sensing mechanisms in PPy derivatives. (**a**) The accompanying contour plots depict Ni-doped PPy (**i**) prior to NH_3_ adsorption and (**ii**) following NH_3_ adsorption [104]. (**b**) Total density of states (TDOS) showing changes in molecular orbital positions, including an increase in the HOMO–LUMO gap following adsorption of NH_3_ on Ni-doped PPy. The TDOS plots compare Ni/PPy (**i**) before ammonia interaction and (**ii**) after ammonia interaction [97].

In contrast, exposure of doped PPy ($PPy^{+}$) to $NH_{3}\;$induces dedoping, while neutral PPy undergoes doping, confirming the $PPy/PPy^{+}$ system as a highly responsive $NH_{3}\;$sensor (Figure 5a) [97]. DFT calculations provide atomic-level support for this mechanism. For instance, the interaction of Ni-doped PPy with NH_3_ leads to measurable changes in the molecular orbital energies, reflected in the TDOS plot shown conceptually in Figure 5b, resulting in an increase in the HOMO–LUMO gap after the interaction [97]. Theoretical studies confirm that PPy exhibits significant adsorption energy towards NH_3_, with the energy increasing slightly as the oligomer chain length increases, stabilizing around 0.479 eV for an infinite chain (nPPy(n = +∞)) [65]. Rad et al. calculated a high BSSE-corrected $NH_{3}\;$interaction energy of 30.58 kJ ${mol}^{-1}$ for a 3-unit PPy model [35,49]. Zhang et al. further confirmed PPy’s selectivity, obtaining an adsorption energy of 0.433 eV for $NH_{3}\;$on PPy nanosheets, significantly larger than for acetone, formaldehyde, or benzene [65]. Experimentally, PPy nanosheets achieved sensitivities of 1.029 and 2.153 at 2 ppm and 500 ppm, respectively, at room temperature. Enhanced performance is often achieved by nanocomposite engineering: NiO–PPy composites reach a maximum response of 0.29 at 225 ppm $NH_{3}$, response/recovery times of 11 s and 18 s, and a limit of detection (LOD) of 17.31 ppm [65]. DFT analysis shows that $NH_{3}\;$adsorption on Ni/PPy increases the band gap from 2.96 to 3.12 eV, indicating localized CT from the gas molecule to the polymer matrix [98,99,100,101,102,103].

For oxidizing gases, structural modification is often necessary to achieve room-temperature sensitivity [44]. Du et al. demonstrated that polarization doping of PPy nanosheets with dodecylbenzenesulfonate ($DBS^{-}$) significantly improves $NO_{2}$ sensing. DFT calculations showed enhanced $NO_{2}\;$adsorption energy, from −0.511 eV for pristine PPy to −0.676 eV for DBS-doped PPy, accompanied by increased electron transfer (from 0.411 ∣e∣ to 0.521 ∣e∣) [44,105]. Wasim et al. also examined oxynitrogen analytes, finding the sensing trend $nPy\;,-\;,NO_{2}^{-}>nPy\;,-\;,NO_{2}>nPy\;,-\;,NO$. These interactions substantially reduce the PPy band gap, down to 2.36 eV for a $1Py\;,-\;,NO_{2}$ complex (vs. 6.87 eV for isolated 1Py) [106].

PPy also enhances sensing of highly oxidative gases such as ozone. Deng et al. developed an IGZO/PPy heterojunction capable of detecting $O_{3}$ at ppb-level concentrations (LOD ≈ 3.9 ppb) at 25 °C without heating or UV illumination [89]. DFT results showed enhanced $O_{3}\;$adsorption energy, from 1.04 eV on IGZO to 1.17 eV on IGZO@PPy, via hydrogen bonding stabilization. The device exhibited a 4-fold sensitivity increase and a 10-fold faster response (from 1077 s to 100 s) relative to pristine IGZO [89].

DFT also informs VOC detection mechanisms. Franco Jr. investigated interactions between a 9-unit PPy model and heterocarbonyl gases (phosgene, formaldehyde, acetone) [57]. The lowest HOMO–LUMO gaps, 2.144 eV for phosgene and 2.171 eV for formaldehyde, indicate high chemiresistive sensitivity [43,57]. Ariyageadsakul et al. found that negatively charged PPy ($nPy^{-}$) binds formaldehyde most strongly; the PPy band gap decreased by 26% (neutral) to 39% (doped ${PPy}^{+}$) upon binding, consistent with high sensitivity [43]. Although $H_{2}O$ shows strong binding (−10.97 to −18.28 kcal ${mol}^{-1}$), both DFT and experiments confirm minimal interference in breath analysis [43].

### 5.2. Electrochemical and Biosensing Applications

PPy-based composites are widely used in electrochemical sensing as their redox activity and conductivity enable rapid electron transfer and strong analyte affinity [80]. DFT calculations guide the rational design of dopants, functional groups, and composite architectures to optimize adsorption energetics and CT pathways.

#### 5.2.1. Electrochemical and Heavy Metal Sensing

Surface functionalization strongly enhances PPy’s electrochemical performance. Wang et al. studied screen-printed graphene electrodes (SPGEs) modified with PPy non-covalently doped by sodium dodecylbenzenesulfonate (SDBS) [80]. DFT revealed that SDBS doping increases the adsorption energy of the ferricyanide probe from −0.41 eV (pure PPy) to −1.11 eV (SDBS/PPy). This enhanced interaction increased CT from 0.40 to 0.56 ∣e∣, improving anodic current sensitivity from 11.38 to 15.17 μA ${mM}^{-1}$. The improvement arises from increased surface area, stability, and catalytic effects resulting from the higher C=O content after functionalization [80].

For heavy metal sensing, PPy’s π-conjugated backbone provides strong affinity for multivalent ions. Lo et al. developed a PPy-coated CNT electrode for $Pb^{2+}\;$detection, achieving a linear analytical range of $10^{-8}\;–\;3\times 10^{-7}\;$mol $L^{-1}$ and an ultralow LOD of 2.9 × $10^{-9}$ mol $L^{-1}$. DFT calculations showed markedly stronger interactions of the chelator EGTA with $Pb^{2+}$(−374.6 kJ ${mol}^{-1}$) than with $Cu^{2+}$(−116.4 kJ ${mol}^{-1}$), explaining the sensor’s excellent selectivity. Similar trends were observed for amino-functionalized graphene-oxide/PPy composites used for $Pb^{2+}$ detection [19].

#### 5.2.2. Biosensing and Molecular Recognition

PPy is widely used in biosensing due to its biocompatibility, ease of functionalization, and compatibility with molecularly imprinted polymers (MIPs) DFT calculations clarify template–polymer interactions and assist in engineering selective binding environments.

Wang et al. investigated SDBS-doped PPy nanosheets on SPGEs for simultaneous detection of dopamine (DA) and uric acid (UA) [96]. DFT results showed that SDBS doping increases DA adsorption energy from 0.034 to 0.184 eV, while CT rises from 0.0729 to 0.1263 ∣e∣. Experimentally, oxidation–current sensitivity increased from 0.08 to 0.13 μA ${\mu M}^{-1}$ for DA and from 0.01 to 0.04 μA ${\mu M}^{-1}$ for UA, with LOD values of 0.076 μM and 0.08 μM, respectively [96].

DFT has also been used to optimize surface chemistry for amino acid detection. A graphene-oxide/PPy/ZnO composite functionalized with carboxyl groups showed the strongest affinity for alanine, producing a decrease in ionization potential (from 3.03 to 2.56 eV) and an increase in electron affinity (from 4.68 to 4.77 eV) [39]. Reduced chemical hardness and increased softness indicated improved electronic reactivity without compromising stability [39].

In MIP-based detection, strong template–polymer interactions underpin selectivity. For glyphosate detection, PPy-MIP films achieved an ultralow LOD of 1 pM, supported by DFT-calculated interaction energies of approximately −145 kJ ${mol}^{-1}$ [102]. Ascorbic acid (AA) sensing using PPy doped with naphthalene sulfonic acid (PPy–NSA) yielded an LOD of 0.55 μM and an LOQ of 1.66 μM, with DFT confirming that van der Waals forces dominate AA–polymer interactions [102,103].

## 6. Environmental Applications

Polypyrrole (PPy) and its nanocomposites have gained substantial attention for environmental remediation, particularly in water purification, pollutant adsorption, and photocatalysis. Their attractiveness stems from PPy’s redox activity, chemical stability, and protonated amino groups, which facilitate adsorption and catalytic interactions [104,105,106,107,108]. DFT plays an essential role in clarifying adsorption mechanisms, charge transfer processes, and pollutant selectivity within PPy-based systems, thereby guiding rational material design. Table 4 summaries DFT and experimental parameters for PPy-based environmental applications.

### 6.1. Pollutant Adsorption and Removal

PPy-containing materials exhibit high efficiency in removing dyes, heavy metals, and emerging contaminants. Incorporating PPy into composite frameworks generally improves surface area, adsorption selectivity, and electron transfer characteristics.

#### 6.1.1. Dyes and Humic Acid By-Products

Tanzifi and colleagues developed PPy/carboxymethyl cellulose (PPy/CMC) nanocomposite particles for the removal of Reactive Red 56 (RR56) and Reactive Blue 160 (RB160). Adsorption followed pseudo-second-order kinetics and the Langmuir isotherm, achieving maximum capacities of 104.9 mg/g for RR56 and 120.7 mg/g for RB160 [35,109]. DFT calculations indicated strong interactions dominated by hydrogen bonding and van der Waals forces, with interaction energies of 85.45 kcal/mol (RB160) and 80.39 kcal/mol (RR56). Adsorption was endothermic [35].

Laabd et al. studied the adsorption of trimellitic acid (TMA) and pyromellitic acid (PMA) on PPy. DFT analysis showed physisorption via hydrogen bonding between the pollutants’ carboxyl groups and PPy’s N–H sites. The process was spontaneous and endothermic, with experimental capacities of 47.62 mg/g (TMA) and 71.43 mg/g (PMA) [110].

#### 6.1.2. Heavy Metals and Emerging Contaminants

PPy’s cationic and redox-active nature enables selective heavy metal removal [111]. Zhang et al. designed montmorillonite (MMT)/PPy composites for Pb^2+^ adsorption [83]. DFT (GGA–PBE with DFT-D2 corrections) revealed 0.41 electrons transferred from PPy to MMT, generating a built-in electric field that enhanced Pb^2+^ binding via Pb–O interactions. The calculated adsorption energy was 4.81 eV. Notably, Pb^2+^ diffusion was predicted to be 5.79 × 10^70^ times faster on the composite than Cd^2+^, explaining kinetic selectivity. Experimentally, the material achieved a capacity of 1345.22 mg/g and retained over 90% efficiency after 10 cycles [83].

He et al. evaluated Cr(VI) removal using Co–PPy-modified layered double hydroxides (CCALP). The system showed a theoretical adsorption capacity of 845.25 mg/g and achieved nearly complete removal (98.83%). DFT and electrostatic potential analyses indicated adsorption driven by electrostatic attraction, ion exchange, and the reduction of Cr(VI) to Cr(III) [111].

Yu et al. explored PPy-coated pyrogenic carbon (PPy@P-BC) for removing PFAS. DFT confirmed a dual mechanism involving electrostatic attraction between PPy’s protonated amino groups and PFAS anions, combined with pore filling. Similarly, metronidazole exhibited spontaneous, exothermic physisorption via O···H–N interactions [105,112].

### 6.2. Photocatalysis and Advanced Oxidation Processes

PPy effectively forms p–n or Z-scheme heterojunctions with semiconductors such as TiO_2_, g-C_3_N_4_, and ZnCdS. These combinations improve visible-light absorption, band gap modulation, and charge separation, enhancing photocatalytic reactions [56,113,114].

#### 6.2.1. Charge Transfer and Band Alignment

Ullah et al. studied PPy/TiO_2_ nanocomposites by modeling pyrrole oligomers (nPy) interacting with Ti_16_O_32_ clusters [113]. DFT revealed strong covalent bonding with interaction energies between −28 and −45 kcal/mol (and up to −72 kcal/mol for longer oligomers). Electron density transfer from PPy to TiO_2_ caused band gap narrowing and enhanced visible-light absorption. Optimal photocatalytic performance was predicted for oligomers containing six to nine pyrrole units [68,113].

In overall water splitting, Butchosa et al. reported synergistic effects between PPy nanoparticles and g-C_3_N_4_. DFT showed the formation of a heterojunction, leading to Fermi level equilibration and charge separation. Negative charge accumulated on g-C_3_N_4_, while PPy carried positive charge, enabling effective proton reduction and water oxidation [114].

#### 6.2.2. Water Molecule Activation

Zhang et al. designed hydrophilic PPy–PSS-coated Zn_x_Cd_1−x_S nanorods (ZCSs) that exhibited improved photocatalytic hydrogen evolution (PHE) [115]. DFT studies showed that PPy and PSS modified the electronic structure of surface sulfur atoms, lowering the energy barrier for water dissociation and stabilizing H intermediates. The optimized composite achieved a PHE rate of 46.1 mmol h^−1^ g^−1^, approximately 8.7 times higher than bare ZCSs [115].

#### 6.2.3. Membrane and Electrochemical Remediation Systems

PPy is widely used in capacitive deionization (CDI) and electrochemical separation due to its conductivity and ion exchange ability [116].

Shen et al. demonstrated that PPy-modified PET membranes efficiently removed nitrate (NO_3_^−^) through electrostatic attraction and ion exchange. DFT confirmed spontaneous, exothermic adsorption, with nitrogen sites on PPy being the primary adsorption centers. Langmuir modeling predicted a maximum capacity of 10.04 mg NO_3_^−^–N/g (equivalent to 45.18 mg NO_3_^−^/g) at 30 °C [117].

PPy-based hybrid electrodes have also shown high potential for Na^+^ removal. DFT studies by Liu and Zhou demonstrated that PPy accelerates Na^+^ migration through pseudocapacitive redox processes while maintaining excellent cycling stability, retaining up to 97% performance over repeated cycles. Additional PPy-based ESIX systems, such as BiOCl/PPy-Cl, exhibit selective chloride extraction, highlighting the versatility of PPy in electrochemical applications [118].

## 7. Limitations, Challenges, Opportunities, and Future Perspectives

While DFT and TD-DFT are widely used to study polymer–substrate interfaces, these methods have well-known systematic limitations. Standard GGA functionals, such as PBE, tend to underestimate band gaps and may inadequately describe weak van der Waals interactions at polymer–substrate interfaces, which can affect the predicted adsorption energies and charge transfer behavior. To mitigate these shortcomings, dispersion-corrected functionals (e.g., PBE-D3, vdW-DF) are commonly employed to better capture π–π stacking and interfacial interactions. Hybrid functionals (e.g., B3LYP, HSE06) improve band gap predictions and electronic localization, though at higher computational cost. Therefore, while quantitative caution is necessary, the combination of dispersion corrections and hybrid functional benchmarks ensures meaningful predictions of PPy–substrate interactions.

The integration of Density Functional Theory (DFT) with experimental investigations has enabled substantial advances in understanding and optimizing polypyrrole (PPy)-based functional composites for energy storage, sensing, and environmental remediation [2]. DFT and time-dependent DFT (TD-DFT) calculations, frequently employing functionals such as UB3LYP/6-31G(d), have elucidated critical, atomic-level modifications in PPy’s tunable electronic structure, particularly the doping and dedoping processes that govern conductivity and charge storage. Computational analyses have reliably modeled the reduction in band gap, shortening of inter-ring bridge lengths, and enhancement in π–electron delocalization upon p-doping, all of which correlate strongly with experimentally observed improvements in conductivity. For PPy-based nanocomposites, DFT simulations have elucidated interfacial phenomena by predicting enhanced binding energies resulting from defects or surface functionalization (e.g., −34 kcal mol^−1^ for PPy on epoxy-functionalized graphene oxide) and quantifying the interfacial charge transfer responsible for the beneficial built-in electric fields [84]. DFT has further elucidated complex kinetic pathways, such as ion migration in MMT/PPy systems for Pb^2+^ adsorption, electron/ion transfer in rechargeable batteries, and bond cleavage/reformation during electrochemical cycling [83]. Strong agreement between theoretical predictions (e.g., band gaps, IR and UV–vis spectra) and experimental measurements confirms the reliability of current computational protocols, reinforcing DFT as a systematic tool for probing microstructural electronic variations and ion migration mechanisms [33,83].

Despite these advances, several intrinsic and practical challenges continue to limit the broad implementation of PPy-based systems. A major limitation is the structural degradation of PPy during extended electrochemical cycling, resulting from volumetric changes associated with polymer chain folding and relinearization [81]. This swelling–shrinkage behavior severely compromises stability in pseudocapacitors and rechargeable batteries [79,81]. Although this disadvantage is common among conjugated polymers, cycle life can be significantly improved when PPy is electrodeposited on nanostructured arrays that distribute mechanical stress more uniformly. The stability of PPy is also strongly influenced by the nature of the charge-balancing ions, as highlighted by Wu et al. A second major constraint is the limited intrinsic conductivity of undoped PPy, which motivates extensive chemical modification or composite formation [85,106]. Moreover, PPy-based actuators and supercapacitors often exhibit inconsistent performance due to complex ion exchange phenomena that depend sensitively on counterions and electrolyte composition, an area that requires further mechanistic investigation [79].

On the computational side, challenges arise in accurately modeling the disordered, amorphous, and high-molecular-weight phases characteristic of real PPy systems [58]. Conventional DFT approaches often underestimate band gaps, motivating the use of hybrid or long-range corrected functionals, albeit at significantly higher computational cost [43]. Slight discrepancies between simulated and experimental vibrational frequencies, as noted by Ullah et al., can originate from differences between gas-phase calculations and condensed-phase measurements, underscoring the difficulty of modeling solvent and thermal effects in realistic electrochemical environments [2]. Additionally, the accurate description of non-covalent interactions, essential for polymer–substrate interfaces, remains computationally demanding; although dispersion-corrected hybrid methods (e.g., M06-2X-D3) perform well, the large size of PPy-based clusters often makes highly accurate wave function theory (WFT) approaches impractical [84]. While this review qualitatively analyzes PPy-based composites, quantitative structure–property relationships (SPR) could be established by combining DFT descriptors with machine learning models. More research efforts should be devoted to the growing potential of Machine Learning Force Fields (MLFFs) and high-throughput screening strategies which have the potential to overcome the inherent timescale and system size restrictions of conventional DFT, particularly for amorphous PPy phases. These ML models, such as Gaussian Approximation Potentials (GAPs), Spectral Neighbor Analysis Potentials (SNAPs), and emerging graph neural network models, could be trained on high-quality DFT datasets to accurately capture the disordered bonding environments, charge delocalization, and dopant–polymer interaction characteristics of amorphous PPy [119,120]. These techniques allow for simulations involving thousands of atoms over nanosecond timescales, allowing for realistic investigation of ion diffusion, structural relaxation, mechanical response, and thermal effects that are inaccessible via classical DFT methods. This approach would predict the effects of doping concentration and functionalization on electronic properties, enabling rational design and optimization of PPy-based materials for energy storage, sensing, and electronic applications.

## 8. Conclusions

This review synthesizes contemporary DFT and TD-DFT findings on PPy-based composites for energy storage, sensing, and environmental remediation. Computational studies employing methods such as UB3LYP/6-31G(d) consistently reproduce key atomic-level modifications, including band gap reduction, contraction of inter-ring bridge lengths, and enhanced π–electron delocalization upon p-doping. For PPy nanocomposites, DFT simulations provide explicit descriptions of interfacial processes, particularly the charge transfer phenomena that generate beneficial built-in electric fields. Enhanced binding energies, such as the −34 kcal mol^−1^ interaction between PPy and epoxy-functionalized graphene oxide—further illustrate the robustness of PPy–substrate interactions. Correspondence between theoretical predictions and experimental results, including band gaps, infrared spectra, and UV–visible absorption features, validates DFT as a reliable method for probing microstructural electronic variation and ion migration mechanisms.

Despite the significant progress enabled by modern computational techniques, several intrinsic and practical challenges continue to hinder the widespread deployment of PPy-based systems. One persistent limitation is the structural degradation of PPy arising from volumetric changes during redox cycling, which diminishes long-term device performance. From a computational perspective, the accurate treatment of disordered, high-molecular-weight, and amorphous PPy phases remains challenging. Conventional DFT approaches often underestimate band gaps, necessitating hybrid or long-range-corrected functionals that impose substantially greater computational demands. Minor discrepancies between simulated and experimental vibrational frequencies, such as those noted by Ullah and co-workers, can typically be attributed to differences between gas-phase calculations and condensed-phase measurements, highlighting the difficulty of accurately representing solvation and finite-temperature effects.

Although this review focuses on atomic-scale DFT simulations the outcomes provide critical guidance for mesoscale modeling and experimental frameworks. In addition to this, trends in electronic descriptors, such as HOMO–LUMO gaps and charge redistribution, can inform conductivity expectations and electrode performance in energy storage devices. By linking atomic-scale insights to experimentally measurable properties, the DFT data serve as a predictive framework that can guide material functionalization, doping strategies, and optimization of composite architectures for practical applications.

Future research should integrate advanced theoretical tools to address these challenges. The adoption of hybrid functionals, such as TD-DFT with M06HF, and the expanded use of ab initio molecular dynamics (AIMD) will improve the modeling of dynamic degradation processes, solvation effects, and decomposition barriers. Opportunities for materials innovation include tailoring PPy properties through atomic-level doping (for example, silicon substitution) and designing advanced hybrid systems (such as MXene@PPy) informed by DFT predictions of band gap modulation and stable charge transfer pathways. Ultimately, stringent theoretical methodology, combined with systematic experimental validation of computed properties, including adsorption energies, diffusion barriers, and density-of-states signatures near the Fermi level, will be crucial for accelerating the rational design of next-generation PPy functional materials.

## Figures and Tables

**Table 1:** Summary of key DFT computational parameters for PPy functional composites in advanced applications.

PPy System Studied	DFT Descriptor	Value (Range)	Application Area	Key Finding	References
PPy/Graphene (GO_3_)	E_a_d_s_, ZPE (M06-2X-D3)	–28.79 kcal/mol	Energy Storage	Strongest physisorption due to epoxy functionalities.	[24]
PPy (Neutral → Cationic)	Eg (UB3LYP/6-31G(d))	3.41 → 2.91 eV	Energy Storage	Doping narrows band gap and promotes planar conductive geometry.	[2]
MMT/PPy Composite	Work Function (Φ)	5.879 eV	Energy Storage	Lower Φ than MMT (7.475 eV), indicating enhanced electron activity.	[23]
∞Py^−^/CH_2_O	Eg Change	0.40 → 1.85 eV	Sensing (CH_2_O)	362.5% sensitivity increase due to band gap enlargement.	[22]
PPy/DBS–NO_2_	E_a_d_s_/Charge Transfer	–0.676 eV/0.521	Sensing (NO_2_)	-	[25]
∞Py/NO_3_^−^ (Aqueous)	Eint (B3LYP-DCP/GEN)	–7.35 kcal/mol	Sensing (NO_3_^−^)	Solvent effects significantly reduce interaction energy.	[3]
9Py^+^ (Polaronic State)	Static Hyperpolarizability (β_0_)	3.2 × 10^4^ au	Optoelectronics	246-fold increase over neutral PPy.	[25]
Si-doped PPy	Eg	Narrowed	Fundamental Tuning	Si-doping yields optimal conductivity enhancement.	[26]

**Table 2:** Representative DFT functionals, parameters, and energetic results for PPy applications.

Functional (XC)	Basis Set/Pseudopotential	System Studied	Key Parameter	Value	Reference(s)
B3LYP/UB3LYP	6-31G(d)	PPy Oligomer (n = 9)	E_9_ (∞Py) Extrapolated	2.88 eV	[43]
M06-2X-D3	6-31G(d)	PPy on Pristine Graphene	E_a_d_s_	−25 kcal mol^−1^	[40]
M06-2X-D3	6-31G(d)	PPy on Graphene Oxide (GO_3_)	E_a_d_s_	−34 kcal mol^−1^	[40]
B3LYP-D3(BJ)-CP	6-31+G(d,p)	1Py–CH_2_O Complex	E_int_ (non-covalent)	−5.74 kcal mol^−1^	[52]
GGA-PBE	PAW/550 eV Cutoff	PPy/SnS Thermoelectrics	Max Force Convergence	0.02 eV Å^−1^	[52]
GGA-PBE	Ultrasoft PP/80 Ry Cutoff	Crystalline PPy-PF_6_	Energy Convergence	10^−8^ Ry unit^−1^ cell	[24]
B3LYP	6-31G(d)	3PPy–CH_4_ Sensor	ΔE_int_, CP	0.11 kJ mol^−1^	[35,49]

**Table 3:** Summary of structure–property correlations.

System or Structural Modification	Property Tuned	DFT Descriptor or Value	Performance Impact	References
PPy (neutral → polaron PPy^+^)	Electronic conductivity	Band gap reduction from 3.41 to 2.91 eV	Insulator-to-conductor transition; planarization and enhanced π-conjugation	[2]
PPy/Graphene Oxide (GO_3_)	Interfacial binding strength	Adsorption energy increased to −34 kcal mol^−1^	Improved mechanical stability through hydrogen bonding and π–π stacking	[40]
PPy/MWCNTs	Electrical conductivity	Optical band gap reduced from 2.89 to 1.58 eV	σdc increased to 56.08 × 10^−5^ S m^−1^	[21]
PPy/MMT	Electroactivity and cation capture	Electron transfer of 0.41 e^−^ (PPy → MMT)	Built-in electric field facilitates Pb^2+^ adsorption; high adsorption energy (4.81 eV)	[51]
PPy/Nanodiamond	Photovoltaic charge transport	HOMO (PPy)/LUMO (ND) spatial separation	Favorable energy alignment for exciton dissociation	[64]
PPy nanostructure (SDBS-modified)	Specific surface area	Diameter reduced from 365 to 148 nm	Increased reaction sites and electrochemical sensitivity	[67]

**Table 4:** Summary of DFT and experimental parameters for PPy-based environmental applications.

System/Pollutant	Key DFT Metric	Value	Experimental Outcome	Reference
MMT/PPy—Pb^2+^	Adsorption energy	4.81 eV	1345.22 mg/g	[83]
MMT/PPy—Pb^2+^	Pb vs. Cd migration rate	5.79 × 10^70^ times faster	>90% retained after 10 cycles	[83]
PPy/CMC—RB160	Interaction energy	85.45 kcal/mol	120.7 mg/g	[35,109,110]
PPy/CMC—RR56	Interaction energy	80.39 kcal/mol	104.9 mg/g	[35,109,111]
PPy—PMA	Mechanism	Hydrogen bond physisorption	71.43 mg/g	[112,113,114,115,116]
PET–PPy—NO_3_^−^	Adsorption type	Spontaneous, exothermic	10.04 mg NO_3_^−^–N/g	[117]
PPy/TiO_2_	Interaction energy	−28 to −45 kcal/mol	Band gap narrowing	[68]
g-C_3_N_4_/PPy	Charge transfer	PPy becomes positively charged	Enhanced water splitting	[114]
PPy–PSS/ZCS	PHE enhancement	8.67× vs. ZCS	46.1 mmol h^−1^ g^−1^	[115]

## Data Availability

Data sharing is not applicable.
